# Editorial: Current trends and strategies for the management of type A aortic dissection

**DOI:** 10.3389/fcvm.2022.1041760

**Published:** 2022-11-08

**Authors:** Giovanni Mariscalco, Metesh Acharya, Varun Shetty, Pradeep Narayan

**Affiliations:** ^1^Department of Cardiac Surgery, Glenfield Hospital, Leicester, United Kingdom; ^2^Department of Cardiac Surgery, Narayana Health, Bengaluru, India

**Keywords:** acute type A dissection, aortic dissection, risk factors, malperfusion, biomarkers

## Introduction

Acute type A aortic dissection (ATAAD) is a life-threatening emergency with a mortality increasing by 1–2% per hour (1, 2). Surgical intervention aims to relieve cardiac tamponade from intra-pericardial aortic rupture, prevent myocardial ischaemia by establishing satisfactory coronary perfusion, resect the primary aortic intimal tear, reconstitute the damaged aorta, restore aortic valve competency, achieve false lumen obliteration and limit propagation of the dissection into branch vessels (3, 4). Patients presenting with ATAAD should therefore be transferred expeditiously to a dedicated aortic center, where the availability of specialist care has been demonstrated to translate into more favorable outcomes in this high-risk population (5). Interestingly, the improved outcome observed in patients with delayed ATAAD presentation who are operated at >48 h beyond symptom onset may reflect their less severe pathology and malperfusion (6).

This Research Topic explores (i) the role of novel biomarkers for prognostication of patients undergoing surgery for ATAAD, (ii) risk factors for post-operative complications and models for their prediction and (iii) current surgical management strategies.

## Biomarkers in acute type A aortic dissection

The careful selection to surgery of patients presenting with ATAAD is of great importance. Biomarkers can facilitate surgical decision-making by discriminating those patients who are more likely to have a better outcome. In this Research Topic, Jiang et al. analyse the association between the pre-operative levels of the biomarker transcription regular protein 1, which linked with the development of ATAAD, and post-operative mortality. Low lymphocyte counts are commonly correlated with organ dysfunction and immunosuppression states, but their role in post-operative surgical outcomes is less well demonstrated. In their original research article, Luo et al. investigate the influence of pre-operative CD4+ lymphopenia on outcomes following ATAAD surgery. These findings will provide greater insight into the role of immunomodulation in ATAAD and by extension stimulate efforts in organ protection during complex aortic surgery. Stroke is one of the most feared neurological adverse events following ATAAD and its surgical repair. Neurofilament Light Chain (NFL) is another promising biomarker being appraised in the post-operative neurological prognosis of patients following surgery for ATAAD, as evaluated by Zhang et al. in another original study within this research theme.

## Risk factors and prediction of outcomes following acute type A aortic dissection

Despite improvements in patient referral pathways, peri-operative care and surgical management, ATAAD continues to carry significant risks of in-hospital mortality. The analysis by Yuan et al. provides valuable information on pre- and intra-operative risk factors affecting mortality in ATAAD patients.

Malperfusion is perhaps the most important risk factor for adverse outcomes following ATAAD with the number of organ systems affected contributing an additive effect on mortality (6). Patients presenting with coma, cerebral or coronary ischaemia and haemodynamic instability are likewise associated with adverse outcomes (7–9). Ischaemic liver injury is prevalent in patients undergoing ATAAD repair on account of the surgical complexity and extended cardiopulmonary bypass and cross-clamp durations. Risk factors for ischaemic liver injury after ATAAD surgery are presented in the study by Liu et al., along with a model for risk calculation in individual patients.

Patients presenting with shock, pericardial tamponade and those requiring cardiopulmonary resuscitation prior to surgery are all associated with adverse outcomes (7). Post-operative bleeding following ATAAD surgery may culminate in irreversible organ dysfunction, and thereby increase mortality. Coinciding with this research theme, important risk factors for massive bleeding after ATAAD surgery, related clinical outcomes and a predictive model are described by Zhang et al. Similarly, Chen et al. report on the various predictors, identified utilizing a machine learning approach, for length of stay on the intensive care unit following surgery for ATAAD. This has important implications for resource allocation and expenditure within critical care settings.

ATAAD additionally carries a relatively high risk of post-operative renal impairment and renal failure is another important risk factor for poor outcome identified in the International Registry of Aortic Dissection database (5). Jiao et al. propose a model to determine in-hospital mortality risk in patients undergoing continuous renal replacement therapy following ATAAD.

Age has a significant bearing on outcome following surgical repair of ATAAD. While mortality in the septuagenarians has been reported to be only 16%, it increases to 35% in octogenarians (9). The incidence of obesity worldwide has increased significantly in recent decades and elevated BMI has been suggested to have a negative impact on outcomes following ATAAD surgery. Pan et al. here examine the relationship between obesity and outcome in a Chinese cohort of patients undergoing surgery for ATAAD.

Earlier studies on gender suggested it to be an important determinant of outcome (10), although more recent studies have shown that gender in the last decade has ceased to be an important risk factor (11, 12). The UK National Adult Cardiac Surgical Audit identified impaired left ventricular function, previous cardiac surgery, need for concomitant coronary artery bypass grafting and preoperative mechanical ventilation as additional risk factors for adverse outcomes (13). The German Registry for Acute Type A Aortic Dissection score in addition found usage of catecholamines at referral, and involvement of arch, head and neck vessels and descending aorta as additional risk factors (14).

## Current surgical management

Surgical intervention for ATAAD involves cardiopulmonary bypass, hypothermic circulatory arrest and retrograde cerebral perfusion (15). Cerebral and myocardial protection require special consideration. Femoral, right axillary and direct aortic cannulation sites for arterial return are variably preferred. Similarly, the optimal perfusion strategy remains debated, with no general consensus regarding the depth of hypothermia for circulatory, and benefits of retrograde vs. selective antegrade cerebral perfusion (16).

The extent of aortic replacement is of paramount importance and determined by the location of intimal tear. Proximally, aortic root replacement may be accomplished using modifications of the Bentall-de Bono procedure. Alternatively, aortic valve preservation with commissural resuspension may be feasible in 60–80% of patients (17) where the valve is structurally normal, or the valve may be directly repaired (18), and combined with reconstruction of the aortic root in the absence of an aortic root aneurysm or aortopathy. In the scenario of extensive aortic root dissection, concomitant aortic valve dysfunction or coronary malperfusion, formal root replacement is mandated alongside potential coronary artery bypass grafting. Carefully selected patients without an aneurysm or tear involving the sinuses of Valsalva may benefit from a valve-sparing root replacement approach to avoid the risks of structural valve degeneration, anticoagulation and endocarditis inherent with prosthetic valve implantation.

Two schools of thought prevail for reconstruction of the aortic arch in ATAAD. Resection of the entry tear can be achieved utilizing a more conservative approach comprising ascending aortic resection with beveled replacement of the underside of the aortic arch (hemi-arch replacement) in nearly all ATAAD cases. A more aggressive operation is reasonable in patients with connective tissue disease or an aneurysmal arch and is essential for intimal tears extending onto the greater curvature of the aortic arch associated with cerebral malperfusion (17). This encompasses complete aortic arch resection with separate direct anastomosis of the supra-aortic vessels into the aortic graft in the conventional elephant trunk procedure, or their reimplantation as a patch. The more contemporary frozen elephant trunk adaptation (18, 19) permits reconstruction of the aortic arch in addition to concurrent stabilization of the descending aorta with deployment of an endovascular stent to address co-existing visceral and limb malperfusion syndromes. Proponents of aggressive arch intervention in ATAAD argue that this strategy enhances long-term survival by encouraging false lumen occlusion, thereby inhibiting descending aortic aneurysm formation and decreasing future reintervention rates on the distal aorta (20, 21). Irrespective of the strategy employed, the distal aortic anastomosis should strictly be performed under circulatory arrest to allow adequate opportunity for careful resection of the dissection aorta and meticulous re-apposition of the aortic wall, thereby preserving cerebral and distal aortic perfusion. With greater emphasis on percutaneous aortic arch intervention in ATAAD, innovative stent-graft designs are being developed. Recently, the Ascyrus Medical Dissection Stent (AMDS) has emerged as a partially uncovered stent permitting continuous arch vessel perfusion and descending aortic coverage (22, 23). In their original research article, Mehdiani et al. present their early results using this prosthesis in ATAAD.

## Conclusion

ATAAD is a devastating aortic complication with high morbidity and mortality necessitating prompt cardiac surgical assessment and intervention. The underlying pathophysiological mechanisms are complex. Just as we begin to uncover and understand more about this entity, there is also great potential in applying this knowledge to improve outcomes for those patients affected by this deadly disease. This broad Research Topic examines current trends and surgical management of ATAAD from both an in-depth scientific and real-world clinical perspective. We hope that the mix of articles provides an enriching experience for the reader and enhances their understanding of this highly important aortic pathology.

## Author contributions

PN and GM contributed to conception and design of the study. PN, VS, GM, and MA wrote the first draft of the manuscript and wrote sections of the manuscript. All authors contributed to manuscript revision, read, and approved the submitted version.

## Conflict of interest

The authors declare that the research was conducted in the absence of any commercial or financial relationships that could be construed as a potential conflict of interest.

## Publisher's note

All claims expressed in this article are solely those of the authors and do not necessarily represent those of their affiliated organizations, or those of the publisher, the editors and the reviewers. Any product that may be evaluated in this article, or claim that may be made by its manufacturer, is not guaranteed or endorsed by the publisher.
